# Effects of intravenous rtPA in patients with minor stroke

**DOI:** 10.1080/07853890.2024.2304653

**Published:** 2024-01-30

**Authors:** Zhihao Lei, Shuanglin Li, Hongye Feng, Xiaohong Wu, Shiyu Hu, Jun Li, Gelin Xu, Lijie Ren, Suyue Pan

**Affiliations:** a Department of Neurology, Nanfang Hospital, Southern Medical University/The First School of Clinical Medicine, Southern Medical University, Guangzhou, China; b Department of Neurology, Shenzhen Second People’s Hospital, First Affiliated Hospital of Shenzhen University Health Science Center, Shenzhen, China; c Shenzhen Cerebrovascular Disease Treatment and Quality Control Center, Shenzhen, China; d Department of Anatomy and Histology, School of Basic Medical Sciences, Shenzhen University Medical School, Shenzhen University, Shenzhen, Guangdong, China

**Keywords:** Thrombolytic therapy, minor stroke, propensity Score-Matched, outcome

## Abstract

**Background:**

Whether minor ischemic stroke (MIS) patients can benefit from intravenous thrombolysis (IVT) remains controversial. The association between the efficacy of IVT and baseline National Institute of Health Stroke Scale (NIHSS) score is unclear in MIS, while the association in moderate and severe stroke is known. This study aimed to explore the effect of IVT in patients with MIS and analyze its efficacy in patients with different baseline NIHSS scores.

**Methods:**

Patients with a NIHSS score ≤5 within 4.5 h of stroke onset were screened in 32 centers. Patients with and without IVT were matched to a ratio of 1:1 with propensity scores. An excellent outcome was defined as a modified Rankin Scale (mRS) score ≤1 at three months after stroke onset. Safety outcomes included mortality and symptomatic intracranial hemorrhage (sICH). Multivariate analysis was used to compute the adjusted odds ratio (OR) for excellent outcomes. The effect of IVT was further analyzed in subgroups according to the baseline NIHSS score.

**Results:**

Of the 23,853 screened, 3336 patients with MIS who arrived at the hospital within 4.5 h of onset were included. The 1163 patients treated with IVT were matched with 1163 patients without IVT. IVT in minor strokes generated an adjusted OR of 1.38 (95% CI: 1.09–1.75, *p* = 0.009) for excellent outcomes. There were no significant differences in mortality (0.17% vs. 0.09%, *p =* 1.000) and sICH (0.69% vs. 0.86%, *p =* 0.813) between patients with and without IVT. Subgroup analysis showed that there was no significant effect of IVT in the baseline NIHSS 0-1 or 2-3 subgroups, with adjusted OR of 0.816 (95% CI 0.437–1.53, *p* = 0.525) and1.22 (95% CI 0.845–1.77, *p* = 0.287), respectively. In patients with NIHSS score of 4–5, IVT was significantly effective, with an adjusted OR of 1.53 (95% CI 1.02–2.30, *p* = 0.038).

**Conclusion:**

IVT can improve MIS outcomes. The risks of sICH and mortality did not increase, especially in patients with NIHSS scores 4 to 5, who could benefit from IVT significantly.

## KEY MESSAGES

- Overall, intravenous thrombolysis can improve the outcomes of patients with minor stroke.
- Minor stroke patients with a baseline NIHSS score of 4-5 can benefit the most from intravenous thrombolysis.

## Introduction

Stroke is the second leading cause of death and third leading cause of disability [1]. Minor ischemic stroke (MIS) accounts for approximately 30% of stroke cases [2]. However, MIS does not necessarily result in favorable outcomes. In an Asian population, the incidence of early neurological deterioration (END) in MIS was reported to be as high as 15.2% [3]. A retrospective study found that 29% of patients with MIS (baseline National Institute of Health Stroke Scale (NIHSS) ≤5 points) who did not receive intravenous (IV) recombinant tissue plasminogen activator antigen (rtPA) treatment had poor outcomes [4]. IV rtPA is currently the first-line treatment for acute ischemic stroke (AIS) within 4.5 h of onset [5]. However, in practice, only 13.5% of MIS were treated with IV rtPA [6,7]. This disadvantageous situation is largely a result of controversy regarding whether patients undergoing MIS could benefit from rtPA treatment. Some studies observed that IV rtPA treatment in patients with MIS was beneficial [8–10]; while others did not [11–13]; while others excluded patients with MIS from their observations [7, 14]. In general, according to the relevant research, the efficacy of IV rtPA may be associated with the baseline NIHSS score in moderate to severe stroke [15], but this relationship remains unclear in MIS patients. The objective of this study was to explore the effect of intravenous rtPA within 4.5 h of onset in MIS patients through a retrospective analysis of registry data and further analyze its efficacy in patients with different baseline NIHSS scores.

## Methods

### Study population

This study followed the Observational Studies in Epidemiology (STROBE) reporting checklist (STROBE statement). Patients were enrolled via a prospective registry that included all 32 advanced stroke centers in Shenzhen City. In this prospective program, all patients with ischemic stroke within 7 days of stroke onset were registered. This study enrolled patients between October 2018 and December 2021. The study was performed in accordance with the Declaration of Helsinki (as revised in 2013) and approved by the Ethics Committee of the Shenzhen Cerebrovascular Disease Treatment and Quality Control Center. Informed consent was waived by the Institutional Review Board.

Patients were included if they (1) were diagnosed with AIS and with onset-to-door time (ODT) ≤4.5 h; (2) were aged 18 years or older; and (3) had a baseline National Institute of Health Stroke Scale (NIHSS) ≤5. Patients were excluded if they (1) had a premorbid modified Rankin Scale (mRS) score >1, (2) were treated with intra-arterial thrombolysis or mechanical thrombectomy, or (3) had severe traumatic brain injury or stroke within 3 months.

### Baseline assessment

Baseline demographic characteristics, including age and sex, and vascular risk factors, including history of AIS, HBP, DM, myocardial infarction, AF, smoking, and alcohol drinking, were self-reported on a questionnaire on admission and verified by a trained neurologist. The baseline NIHSS and premorbid mRS scores on admission were also assessed. Trained nurses measured baseline systolic blood pressure (SBP) and baseline diastolic blood pressure (DBP) on admission. The stroke physician confirmed the diagnosis of AIS and classified the cases according to the modified TOAST (Trial of ORG 10172 in Acute Stroke Treatment) [16]. Trained neurologists provided standardized care to patients according to the recommendations of the guidelines [5] and decided whether to administer intravenous thrombolysis to patients.

### Follow-up assessment

The mRS was used by trained neurologists to assess the functional outcome at 3 months (90 ± 7 days) after onset, ranging from 0–6, with 0 indicating no disability and 6 indicating mortality. We conducted a telephone interview if the patient could not be present in the hospital for assessment. Patients lost to follow-up were excluded from further analysis. Since the symptoms of neurological deficits in the included patients were mild, we defined an mRS ≤1 at 3 months after onset as an excellent outcome. Safety outcomes included mortality and symptomatic intracranial hemorrhage (sICH). sICH was defined using the European Cooperative Acute Stroke Study 3 protocol [7].

### Propensity score matching

**Figure 1.**
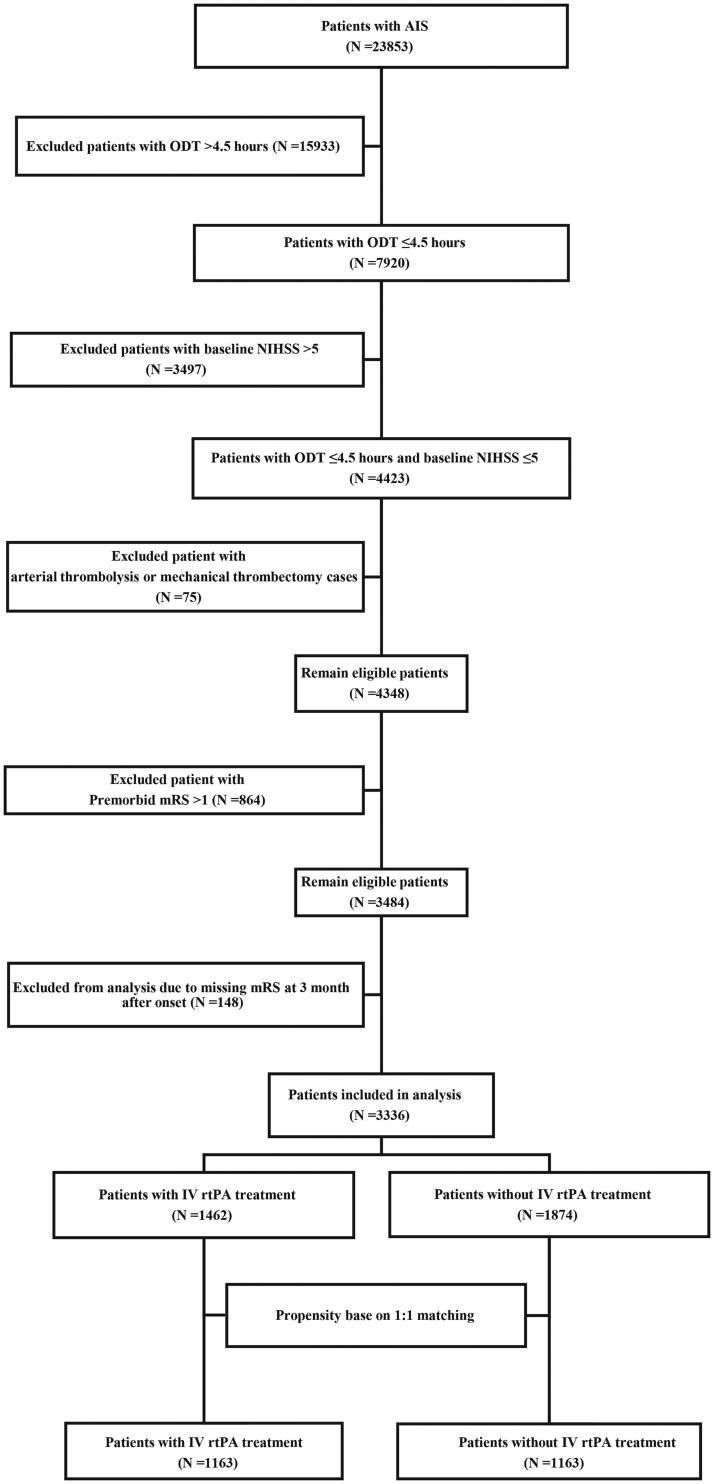
Flowchart of included patients.

Propensity Score (PS) was calculated to estimate the probability of each patient based on a multivariate logistic regression model. The model included nine PS-matched variables: age; sex; premorbid mRS score; baseline NIHSS score; and history of AIS, HBP, DM, myocardial infarction, and AF. We matched patients who received IV rtPA treatment with controls in a 1:1 ratio within a 0.2 times SD of the logit of PS using nearest neighbor matching according to the caliper method. Figure 1 shows the flow diagram of the participant selection process. Online Supplementary Figure S1 presents the distribution of propensity scores, assessing the balance between the two groups before and after matching. In addition, standardized differences in the mean (SMD) were used to estimate the balance between the cases and controls in the cohort without PS matching and the PS-matching cohort, assessing the quality of the matching. SMD >0.1 was deemed as imbalance.

### Statistical analysis

Continuous variables are expressed as mean ± SD or median (IQR), and categorical variables are expressed as frequencies. Comparisons were performed between patients with and without IV rtPA treatment according to baseline characteristics and outcomes in the cohort without PS- and PS-matched cohorts, respectively. Missing data in the PS-matching cohort were handled using multiple imputation (MI) with a regression-switching approach (chained equations with *m* = 10). The distribution of variables with missing data before and after MI was presented. To assess the relationship of IV rtPA treatment with outcome, baseline characteristics with SMD values >0.1 were included in multivariable regression analyses. In addition, we further analyzed demographics, stroke severity, and risk factors, including age, sex, baseline NIHSS score, premorbid mRS score, history of HBP, DM and AF, and smoking and alcohol consumption in the model, regardless of their SMD values. Sensitivity analyses were performed in the PS-matched + MI cohort and confirm the results. We conducted subgroup analyses to find potential factors that may interact with the efficacy of IVT, including sex, risk factors, etiology, baseline NIHSS score, etc. To analyze the effect of intravenous thrombolytic therapy in MIS with different baseline NIHSS scores, the patients were divided into three subgroups based on baseline NIHSS scores: 0–1, 2–3 and 4–5 groups. PS matching was performed in each subgroup to minimize the influence of baseline imbalances. Multivariate regression analyses adjusting for characteristics with the value of SMD >0.1 were conducted to calculate the adjusted odds ratios (ORs) (95% CI) in each group before and after the subgroup PS matching. The distributions of mRS at 3 months in each subgroup are presented graphically. Statistical analyses were performed using R version 4.1.3 (http://www.r-project.org/). All statistical tests were two-tailed, and *P* values less than.05 were considered statistically significant.

## Results

A total of 23,853 patients with AIS within 7 days of onset were admitted to 32 centers in Shenzhen between October 1, 2018, and December 31, 2021. Among them, 4423 patients with MIS reached the emergency department within 4.5 h of onset, 3484 patients met the eligibility criteria, and 148 patients were lost to follow-up. Finally, 3336 patients were included in the analysis, of whom 1462 (43.8%) received IV rtPA treatment (Figure 1). The differences in the baseline characteristics between those who were included and those who were lost to follow-up were shown in online Supplementary Table S1. The proportions of patients receiving IV rtPA treatment in the two groups were 43.8% and 45.9%, respectively, with no significant difference. In patients included in the analysis, the absolute SMD values for age, baseline NIHSS score, premorbid mRS score, History of AIS, TOAST factors were >0.1. The largest SMD was 0.610 (baseline NIHSS, online Supplementary Table S2).

Using the PS-matched method, 1163 patients with IV rtPA treatment were matched to 1163 patients without IV rtPA treatment (*n* = 2326), which was the PS-matched cohort. The unmatched 1010 patients were excluded from the PS analysis (Figure 1). Measures of balance diagnosis implied that the imbalances in the distribution of PS scores between groups were rectified after PS matching (online supplemental, online Supplementary Figure S1). However, there were still differences in the SMD for TOAST between the groups (SMD >0.1, Table 1).

**Table 1.** Baseline characteristic and outcomes in patient cohort of PS-matching.

Characteristics	Patients without IV rtPA treatment (*n* = 1163)	Patients with IV rtPA treatment (*n* = 1163)	*P*	SMD
Age, year, mean (SD)	59.1 (13.8)	59.1 (12.7)	.995^d^	<0.001
Male, *n* (%)	856 (73.6)	852 (73.3)	.888^a^	0.008
Myocardial infarction, *n* (%)	20 (1.70)	23 (1.98)	.758^a^	0.019
History of AIS, *n* (%)	179 (15.4)	180 (15.5)	1.000^a^	0.002
Baseline NIHSS, median (IQR)	2 (1, 4)	2 (1, 3)	.836^a^	0.012
0–1, *n* (%)	369 (31.7)	346 (29.8)		
2–3, *n* (%)	499 (42.9)	570 (49.0)		
4–5, *n* (%)	295 (25.4)	247 (21.2)		
Alcohol consumption, *n* (%)	192 (16.6)	216 (18.6)	.172^a^	0.060
Smoking, *n* (%)	411 (35.3)	403 (34.7)	.832^a^	0.011
Baseline SBP, mmHg, mean (SD)	150 (23.9)	150 (21.9)	.424^d^	0.033
Baseline DBP, mmHg, mean (SD)	89.7 (15.2)	89.2 (14.8)	.448^d^	0.032
Premorbid mRS =1 (*n*, %)	361 (31.0)	345 (29.7)	.499*	0.030
TOAST			.002^a^	**0.175**
LAA, *n* (%)	517 (44.5)	461 (39.6)		
SVD, *n* (%)	429 (36.9)	458 (39.4)		
CE, *n* (%)	64 (5.50)	53 (4.60)		
ODE, *n* (%)	42 (3.60)	63 (5.42)		
UDE, *n* (%)	45 (3.90)	75 (6.45)		
HBP, *n* (%)	842 (72.4)	835 (71.8)	.781^a^	0.013
DM, *n* (%)	302 (26.0)	310 (26.7)	.742^a^	0.016
AF, *n* (%)	31 (2.70)	35 (3.00)	.708^a^	0.021
sICH, *n* (%)	10 (0.86)	8 (0.69)	.813^a^	0.020
Hospital stays, median (IQR), days	8.26 (5.84, 11.7)	7.98 (5.89, 11.0)	.661^c^	0.023
Mortality, *n* (%)	1 (0.09)	2 (0.17)	1.000^b^	0.024
Excellent outcome, *n* (%)	971 (83.5)	1010 (86.8)	.027^a^	0.094

^a^ Pearson’s χ^2^ test.

^b^ Fisher’s exact test.

^c^ Mann–Whitney U test.

^d^ *t*-test.

PS: Propensity score; IV: intravenous; rtPA: recombinant tissue plasminogen activator antigen; SMD: standardized differences of the mean; AIS: acute ischemic stroke; NIHSS: National Institute of Health Stroke Scale; HbA1c: hemoglobin A1c; FBG: fasting blood glucose; HCY: homocysteine; SUA: serum uric acid; SBP: systolic blood pressure; DBP: diastolic blood pressure; mRS: modified Rankin Scale; TOAST: Trial of ORG 10172 in Acute Stroke Treatment; LAA: large artery atherosclerosis; SVD: small vessel disease; CE: cardio-embolism; ODE: other determined etiology; UDE: undetermined etiology; HBP: hypertension; DM: diabetes mellitus; AF: atrial fibrillation; sICH: symptomatic intracranial hemorrhage.

**Figure 2.**
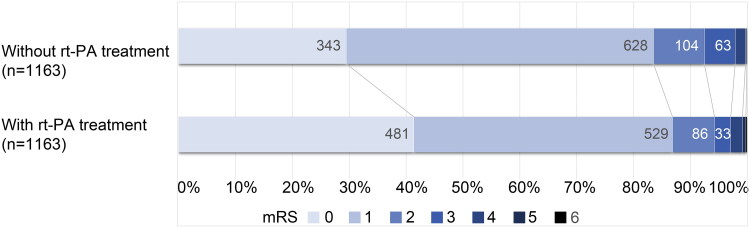
Distributions of mRS at 3 months of onset in the cohort of propensity score-matching.

In the cohort without PS matching, the rate of excellent outcome in patients with IV rtPA treatment was lower than in those without IV rtPA treatment (85.2% vs. 87.6%), but the baseline NIHSS score was significantly higher (3 (1, 4) vs. 1 (0, 3), *p <* .001). In the PS-matched cohort, the proportion of patients with excellent outcomes after IV rtPA treatment was higher (86.8% vs. 83.5%). There was no significant difference in the baseline NIHSS score between the groups (2 (1, 4) vs. 2 (1, 3), *p* = .836, Table 1). The full distribution of disability levels on the mRS score at three months after onset is shown in Figure 2. As for safety outcomes, the mortality and sICH rates of the patients with IV rtPA treatment in the cohort without PS-matching were 0.20% and 1.20%, respectively (Online Supplementary Table S2). The incidence of these adverse events was very low and, more importantly, in the PS-matched cohort, there were no significant differences in sICH (0.69% vs. 0.86%, *p* = .813) or mortality (0.17% vs. 0.09%, *p* = 1.000) between the groups with and without IV rtPA treatment (Table 1). IV rtPA treatment did not extend the hospital stay (7.98 (5.89, 11.01) vs 8.26 (5.84, 11.7), *p =* .661, Table 1). Missing data in the PS-matched cohort were treated with MI, generating a cohort of PS-matched + MI patients. The distribution of variables with missing data before and after MI is shown in online Supplementary Table S3. The results did not change significantly after imputation.

**Figure 3.**
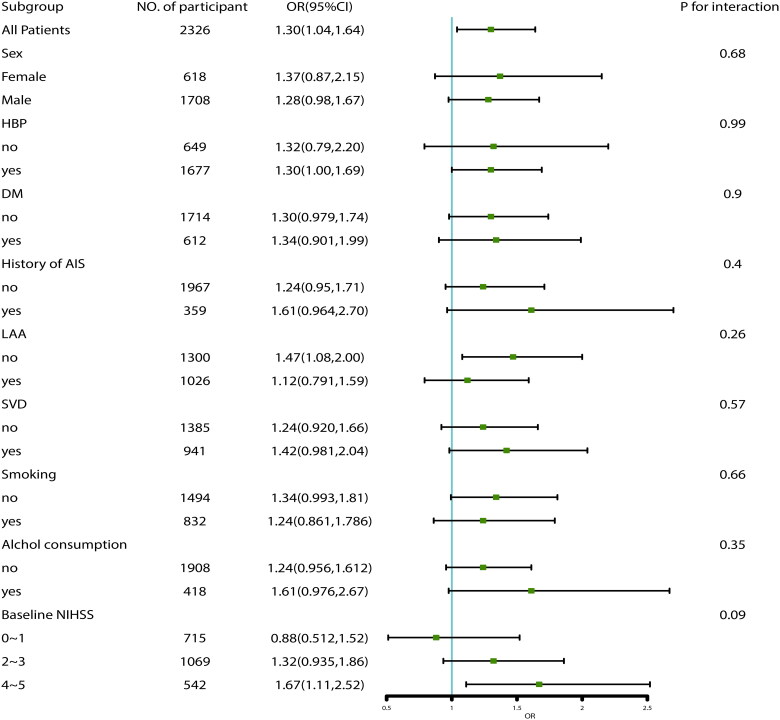
Subgroup analyses of associations between IV-tPA treatment and functional outcome in the cohort of PS-matched + MI.

We conducted multivariate analysis in the cohorts with and without PS-matched (Table 2). In cohort of PS-matching, IV rtPA treatment and variables with SMD >0.1, including TOAST factors, were included in model 1. The results showed that rtPA treatment was superior to standard care without rtPA treatment, with an odds ratio (OR) of 1.38 (95% confidence interval [CI] 1.09–1.75; *p =* .009). We further subjected demographics, stroke severity, and risk factors, including age, sex, baseline NIHSS score, premorbid mRS score, history of HBP, DM, and AF, and smoking and alcohol drinking into the model, regardless of their SMD values (Model 2), which showed similar results (OR =1.45, 95% CI 1.12 to 1.87; *p =* .004). In cohort without PS-matching, Model 3 adjusted for baseline NIHSS, premorbid mRS, age, history of AIS and TOAST factors generated an OR of 1.32 (95% CI 1.04 to 1.66; *p =* .022); Model 4 adjusted for baseline NIHSS, premorbid mRS, age, sex, TOAST, history of AIS, HBP, DM, AF, smoking and alcohol drinking factors, generated an OR of 1.29 (95% CI 1.02 to 1.65; *p =* .036). Sensitivity analyses were conducted in cohort of PS-matching + MI to verify the reliability of the results. Model 5 adjusted for TOAST factors, revealed an OR of 1.30 (95% CI 1.04 to 1.64; *p =* .024), Model 6 adjusted for age, sex, baseline NIHSS score, premorbid mRS score, history of HBP, DM, and AF, and smoking, alcohol drinking and TOAST factors, revealed an OR of 1.38 (95% CI 1.08 to 1.76; *p =* .009). We further performed subgroup analyses in the PS-matching + MI cohort adjusting for TOAST factors (Figure 3), showing that no significant interaction factors with the efficacy of rt-PA treatment were found (*P* for interaction >.05).

**Table 2.** Odd rates for excellent outcome according to IV rtPA treatment in patients with minor stroke.

	Adjusted OR (95% CI)	*P*
Cohort of PS-matching		
Model 1	1.38 (1.09–1.75)	**.009**
Model 2	1.45 (1.12–1.87)	**.004**
Cohort without PS-matching		
Model 3	1.32 (1.04–1.66)	**.022**
Model 4	1.29 (1.02–1.65)	**.036**
Cohort of PS-matching and MI		
Model 5	1.30 (1.04–1.64)	**.024**
Model 6	1.38 (1.08–1.76)	**.009**

Model 1 was adjusted for TOAST factors. Model 2 was adjusted for baseline NIHSS, premorbid mRS, age, sex, history of HBP, DM, AF, smoking, alcohol drinking, and TOAST factors. Model 3 adjusted for baseline NIHSS score, premorbid mRS score, age, history of AIS, and TOAST factors. Model 4 was adjusted for baseline NIHSS, premorbid mRS, age, sex, history of AIS, HBP, DM, AF, smoking, alcohol drinking, and TOAST factors. Model 5 was adjusted for TOAST factors. Model 6 adjusted for baseline NIHSS score, premorbid mRS score, age, sex, history of HBP, DM, AF, smoking, alcohol drinking, and TOAST factors.

Abbreviations: OR, odds ratio; CI, confidence interval; IV, Intravenous; rtPA, recombinant tissue plasminogen activator antigen; PS, propensity score; MI, multiple imputation; TOAST, Trial of ORG 10172 in Acute Stroke Treatment; LAA, large artery atherosclerosis; mRS, modified Rankin Scale; HBP, hypertension; DM, diabetes mellitus; AF, atrial fibrillation; AIS, acute ischemic stroke.

**Figure 4.**
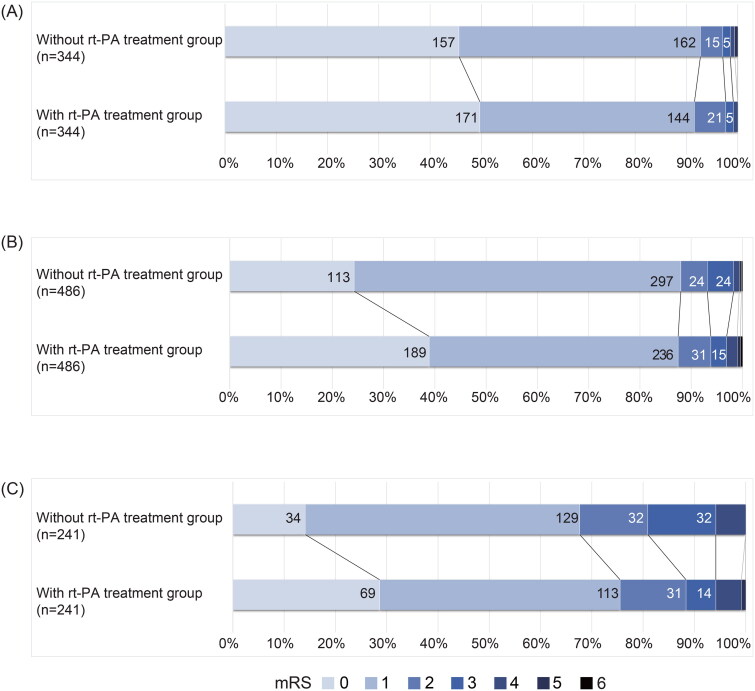
Functional outcomes assessed by mRS among patients with MIS in each subgroup. (A) Functional outcomes among patients with an NIHSS of 0–1. (B) Functional outcomes among patients with an NIHSS of 2–3. (C) Functional outcomes among patients with an NIHSS of 4–5.

Patients included in the analysis were divided into three subgroups based on their baseline NIHSS score: 0–1, 2–3 and 4–5. Baseline characteristics of the subgroup patients before and after matching are shown in online Supplemental Table S4–S6. Variables with SMD> 0.1 were included in the multivariate analysis. Table 3 shows the unadjusted and adjusted ORs of each group, implying effects of greater on efficacy of rtPA treatment with higher baseline NIHSS, with an adjusted OR of 0.816 (95% CI 0.437 to 1.53, *p* = .525) in the 0–1 group, with an adjusted OR of 1.22 (95% CI, 0.845 to 1.77, *p* = .287) in the 2–3 group and adjusted OR of 1.53 (95% CI 1.02 to 2.30], *p* = .038) in the 4–5 group for excellent outcome. Figure 4 shows the functional outcomes 3 months after stroke onset in each subgroup.

**Table 3.** Odd rates for excellent outcome according to IV rtPA treatment in each subgroup with different baseline NIHSS score.

Subgroup		Patients with IV rtPA treatment, n (%)	Unadjusted OR (95%CI)	*P*	Adjusted OR (95%CI)	*P*
Baseline NIHSS 0-1 group	Before PS matched	366, 27.5%	0.626 (0.392-0.999)	**0.050**	0.844 (0.482-1.476)^a^	0.552
	After PS matched	344, 50.0%	0.851 (0.488-1.486)	0.571	0.816 (0.437-1.526)^b^	0.525
Baseline NIHSS 2-3 group	Before PS matched	666, 52.7%	1.108 (0.810-1.515)	0.520	1.07 (0.772-1.494)^c^	0.670
	After PS matched	486, 50.0%	1.29 (0.898-1.857)	0.168	1.22 (0.845-1.771)^d^	0.287
Baseline NIHSS 4-5 group	Before PS matched	430, 57.9%	1.443 (1.032-2.017)	**0.032**	1.65 (1.145-2.370)^e^	**0.007**
	After PS matched	241, 50.0%	1.48 (0.991-2.199)	**0.056**	1.53 (1.024-2.296)^f^	**0.038**

^a^ Indicated adjustment for history of AIS, baseline NIHSS, baseline SBP, DM, and TOAST factors.

^b^ Indicated adjustment for TOAST factors.

^c^ Adjustment for history of AIS, baseline NIHSS, baseline SBP, HBP, and TOAST factors.

^d^ Adjusted for age, DM, and TOAST factors.

^e^ Adjusted for age, sex, history of AIS, baseline NIHSS, baseline DBP, and TOAST factors.

^f^ Indicated adjustment for baseline NIHSS and TOAST scores.

OR: odds ratio; CI: confidence interval; IV: Intravenous; rtPA: recombinant tissue plasminogen activator antigen; PS: propensity score; TOAST: Trial of ORG 10172 in Acute Stroke Treatment; LAA: large artery atherosclerosis; mRS: modified Rankin Scale; HBP: hypertension; DM: diabetes mellitus; AF: atrial fibrillation; AIS: acute ischemic stroke.

## Discussion

This study found that, intravenous thrombolytic therapy improves outcomes in minor strokes, and patients with NIHSS 4–5 count for the benefit not all MIS patients. Actually, no benefit of thrombolysis over no thrombolysis was seen in patients with NIHSS 3 or less.

Some studies have reported the efficacy of IV rtPA treatment in MIS compared with standardized treatment without IV rtPA treatment, and most of these studies provided insufficient evidence owing to the small sample size [11, 17]. In a meta-analysis study in 2018, You et al. partially overcame the disadvantage of a small sample size. A total of 1591 subjects were included in seven studies, among which 801 (50.35%) patients received intravenous thrombolysis. The study suggested a high odd of excellent outcome based on the mRS 0–1 (OR =1.43; *p* = .002) in patients treated with rtPA compared to those without rtPA [10]. Another retrospective analysis from the Third China National Stroke Registry (CNSR-III) included 1905 MIS patients, 527 (28%) of whom received IV rtPA treatment [18]. The results showed that IV rtPA was associated with favorable functional outcomes at 3 months (OR =1.51; 95% CI 1.09 to 2.10; *p* = .01), which was in line with our study.

Previous study suggested that the efficacy of IV rtPA may be associated with the baseline NIHSS score, with severe patients benefiting more than moderate patients (*P* for interaction = 0.003) [15]. Recent studies have focused on this association in patients with MIS. A real-world study included 703 patients with IV rtPA treatment with a baseline NIHSS of 0–1, compared the outcomes of 6316 patients with IV rtPA treatment with a baseline NIHSS of 2–5. The results showed that in the NIHSS 0–1 group, IV rtPA treatment was associated with a lower rate of excellent outcome (adjusted OR =0.67). In the NIHSS 2–5 group, IV rtPA treatment was associated with a higher rate of excellent outcomes (adjusted OR = 1.21, CI 1.08–1.34)[19]. Another study in 2021 included 1765 patients with MIS and TIA who arrived at the hospital within 4.5 h after onset. The effect of IV rtPA treatment on outcomes was not identified in the NIHSS 0–2 subgroup (OR =0.88, *p >* .05), while a suggestion of efficacy was noted in the NIHSS 3–5 subgroup (OR =1.28; *p <* .05) [20]. These studies suggest that IV rtPA treatment is more inclined to improve outcomes in MIS patients with higher baseline NIHSS scores. In the latest controlled clinical trial (RCT) study, ARAMIS study, the subgroup analysis also indicated the similar trend, with a risk difference (RD) of −2.6 (95% CI −8.7 to 1.4) in baseline NIHSS 4–5 group, less than 2.9 (–1.3 to 7.2) in NIHSS 1–3 group and 6.9 in NIHSS 0 group [13]. These results were in accordance with those of our study. In addition, we found no significant effect of intravenous thrombolysis in patients with baseline NIHSS 3 or less in our study. Previous research also showed similar result. Greisenegger et al. detected a significant benefit in favor of rt-PA treatment in patients with baseline NIHSS = 4 or 5 (OR = 1.49; 95% CI: 1.17 to 1.89; *p* < .001). For patients with baseline NIHSS ≤3, they did not detect a significant effect of rt-PA treatment [21]. It may indicate that thrombolytic therapy is not highly recommended for minor strokes with a baseline NIHSS score of 3 or less.

However, some studies have obtained different results on the effects of rtPA treatment in patients with minor strokes. The study by Huisa et al. included 133 patients with MIS who arrived at the hospital within 3 h of onset, 59 (44.36%) of whom received IV rtPA treatment, showing that patients who received IV rtPA treatment had similar outcomes to those who did not receive IV rtPA treatment (OR = 0.93; *p* = .87) [22]. The sample size was small, and baseline NIHSS scores were significantly higher in the IV rtPA treatment group than in the group without IV rtPA treatment (3.40 ± 1.40 vs 1.90 ± 1.30, *p* < .001), which may explain the lack of benefit in patients treated with rtPA. The PRISMS study was the first RCT published [12]. The study randomized 313 subjects, 156 (49.8%) of whom underwent IV rtPA treatment, and concluded that IV rtPA treatment did not increase the probability of good functional outcomes compared with aspirin therapy without IV rtPA treatment. Unfortunately, the study was terminated early owing to other reasons, resulting in an insufficient number of participants, which would interfere with the results to some extent. Another RCT, the ARAMIS study, included 760 patients with acute minor nondisabling stroke, 350 (46.1%) of whom received IV rtPA treatment, showing that dual antiplatelet therapy was noninferior to intravenous alteplase [13]. However, both RCTs enrolled predominantly very mild patients, with only 47 (15.0%) and 134 (17.6%) of the subjects having baseline NIHSS score of 4–5 in the PRISMS and ARAMIS studies, respectively. In comparison, our study and the retrospective study from CNSR-III [18] included a higher proportion of patients with baseline score of 4–5, 23.3%, and 27.4%, respectively. Patients with higher baseline NIHSS scores could benefit more from IV rtPA treatment. This may explain why the RCTs reached different conclusions.

In the clinical setting, the risk of sICH is one of the major reasons why patients with MIS are excluded from IV rtPA treatment. According to previous studies, the probability of sICH in patients with MIS treated with rtPA was in the range of 0% to 2.50% [21, 23], which is lower than that reported in AIS trials in general [24]. In our original cohort (cohort without PS matching), we found that the incidence of sICH was approximately 1.20% in patients receiving IV rtPA treatment (online Supplemental Table S2), which was similar to previous studies. Moreover, in the PS-matched cohort, after reducing the effect of imbalance between the two groups, there were no significant differences in sICH (0.70% vs. 0.90%, *p =* 0.813) or mortality (0.20% vs. 0.10%, *p =* 1.000) between the two groups with and without IV rtPA treatment, as shown in Table 1. These consistent results indicated that IV rtPA treatment in patients with MIS was safe.

However, some limitations of this study should be addressed when interpreting the results. First, the study was based on a prospective registry database. Some important confounders, such as Alberta Stroke Program Early CT (ASPECT) score, time point of start of IVT, and collaterals, etc., did not collected. These may lead to inevitable imbalances in the baseline characteristics between the groups. Although we attempted to minimize the influence of baseline imbalances through the propensity scoring method, it could not substitute for a randomized clinical trial (RCT) to demonstrate the efficacy of IV rtPA treatment in patients with MIS. Second, in most MIS studies, the authors defined MIS based on the baseline NIHSS score. The NIHSS is a convenient tool for selecting IV rtPA treatment candidates in clinical settings, and so was our study. However, in this way, we lacked information on NIHSS sub-items (aphasia, hemianopsia, consciousness, motor, etc.), which prevented us from differentiating nondisabling from disabling patients. Third, due to the lack of RCT data clinicians assign greater weight to motor and speech/language deficits than other neurological deficits, patient age, relative contraindications to thrombolysis, and premorbid disability when deciding to thromboses patients with minor stroke [25]. This may cause bias in the NIHSS score and interfere in the selection of patients for thrombolysis to some extent. Fourth, our study did not collect information about large vessel occlusion (LVO), making it impossible to analyze the effect of IVT in minor strokes with LVO. Previous retrospective study showed minor strokes with LVO may benefit from IVT significantly [26]. Seners et al. [27] conducted a study found that, compared with IVT alone, bridging therapy (IVT followed by endovascular treatment) was not associated with excellent outcome. However, bridging therapy may be beneficial in M1 occlusions, whereas the benefit–risk profile may favor IVT alone in M2 occlusions among minor strokes with LVO. These should be further investigated in RCTs. Finally, this study enrolled patients from 32 centers in Shenzhen. Although this is an immigrant city, and the residents came from all over the country, this result may not be generalizable to other populations. Overall, the findings of this study have to be viewed as hypothesis generating.

## Conclusions

This study found that, overall, IV rtPA treatment can improve MIS outcomes. The risk of sICH and mortality did not significantly increase, and hospital stay was not extended. However, patients with NIHSS 4-5 count for the benefit not all MIS patients. In fact, we found no significant effect of IVT in patients with baseline NIHSS 3 or less.

## Supplementary Material

Supplemental Material

## Data Availability

The data analyzed in the current study are available from the corresponding author upon reasonable request.
