# Supplementary material for: Effects of intravenous rtPA in patients with minor stroke
Source: Ann Med. 2024 Jan 30;56(1):2304653. doi: 10.1080/07853890.2024.2304653 (PMC10829835; doi:10.1080/07853890.2024.2304653)
Supplement: Supplemental Material [file IANN_A_2304653_SM4659.doc]

**SUPPLEMENTAL MATERIAL**

**Title: Feasibility of Intravenous rtPA in Patients with Minor Stroke in the Real World**

| List | Title | Page |
| --- | --- | --- |
| Figure S1 | Distribution of propensity scores and balance measures between groups with and without IV rtPA treatment, in the cohort before and after PS-matching, respectively | 2 |
| Table S1 | Baseline characteristic among patients who were included and those who were lost of follow-up. | 3, 4 |
| Table S2 | Baseline characteristic and outcome of patient in cohort without PS-matching | 5, 6 |
| Table S3 | Distribution of variables with missing data before and after MI | 7 |
| Table S4 | Baseline characteristic and outcome of patients before and after PS matched in subgroup of baseline NIHSS 0- 1. | 8, 9 |
| Table S5 | Baseline characteristic and outcome of patients before and after PS matched in subgroup of baseline NIHSS 2- 3. | 10, 11 |
| Table S6 | Baseline characteristic and outcome of patients before and after PS matched in subgroup of baseline NIHSS 4- 5. | 12, 13 |

**Figure S1.** Distribution of propensity scores and balance measures between groups with and without IV rtPA treatment, in the cohort before and after PS-matching, respectively


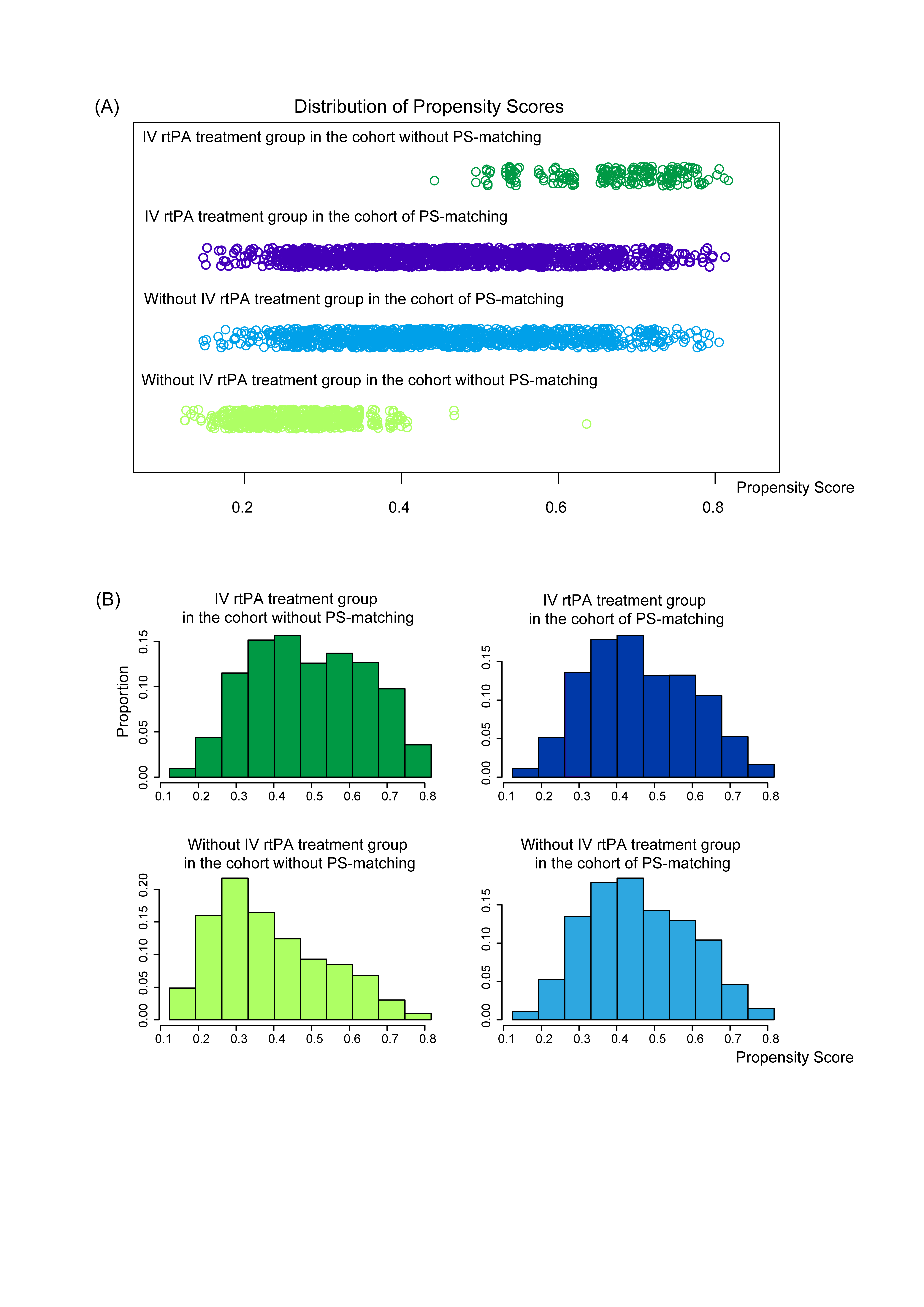


**Table S1. Baseline characteristic among patients who were included and those who were lost of follow-up.**

| Characteristics | Included Patients  (n =3336) | Patients Lost to Follow Up  (n =148) | *P* value | SMD |
| --- | --- | --- | --- | --- |
| Age, year, mean (SD) | 59.5(13.3) | 59.2 (12.2) | <0.819^d^ | 0.020 |
| Male, n (%) | 2442 (73.2) | 108 (73.0) | 1.0000^a^ | 0.006 |
| Myocardial infarction, n (%) | 60 (1.80) | 0 (0.00) | 0.177^b^ | 0.192 |
| History of AIS, n (%) | 560 (16.8) | 10 (6.90) | **0.002^a^** | 0.311 |
| Baseline NIHSS, median (IQR) | 2 (1, 3) | 2 (1, 3) | 0.056^c^ | 0.179 |
| 0-1, n (%) | 1330 (39.9) | 62 (41.9) |  |  |
| 2-3, n (%) | 1263 (37.9) | 65 (43.9) |  |  |
| 4-5, n (%) | 743 (22.3) | 21 (14.2) |  |  |
| Alcohol drinking, n (%) | 577 (17.8) | 27 (18.9) | 0.832^a^ | 0.027 |
| Smoking, n (%) | 1178 (35.0) | 47 (33.1) | 0.458^a^ | 0.072 |
| Baseline SBP, mmHg, mean (SD) | 151 (23.1) | 149 (24.7) | 0.384^d^ | 0.071 |
| Baseline DBP, mmHg, mean (SD) | 89.5 (15.0) | 89.8 (14.0) | 0.809^d^ | 0.021 |
| Premorbid mRS =1(n, %) | 1009 (31.9) | 36 (26.3) | 0.196^a^ | 0.124 |
| TOAST |  |  | 0.105^a^ | 0.225 |
| LAA, n (%) | 1338 (42.7) | 48 (34.0) |  |  |
| SVD, n (%) | 1337 (42.6) | 64 (45.4) |  |  |
| CE, n (%) | 157 (5.0) | 10 (7.10) |  |  |
| ODE, n (%) | 139 (4.4) | 6 (4.30) |  |  |
| UDE, n (%) | 165 (5.30) | 13 (9.20) |  |  |
| HBP, n (%) | 2402 (72.0) | 68 (45.9) | **<0.001^a^** | 0.549 |
| DM, n (%) | 885 (26.5) | 43 (29.1) | 0.559^a^ | 0.056 |
| AF, n (%) | 86 (2.60) | 4 (2.70) | 0.792^b^ | 0.008 |
| sICH, n (%) | 28 (0.80) | 3 (2.00) | 0.143^b^ | 0.100 |
| Hospital stays, median (IQR), days | 7.96 (5.78, 11.1) | 7.00 (4.64, 9.46) | **<0.001^c^** | 0.196 |
| IV rtPA treatment | 1462 (43.8) | 68 (45.9) | 0.671^a^ | 0.043 |

^a^ Pearson’s χ^2^ test, ^b^ Fisher’s exact test, ^c^ Mann-Whitney U test, ^d^ t-test.

Abbreviation: PS, Propensity scores; IV, Intravenous; rtPA, recombinant tissue plasminogen activator antigen; SMD, standardized differences of the mean; AIS, acute ischemic stroke; NIHSS, National Institute of Health Stroke Scale; SBP, systolic blood pressure; DBP, diastolic blood pressure; ; mRS, modified Rankin Scale; TOAST, Trial of ORG 10172 in Acute Stroke Treatment; LAA, large artery atherosclerosis; SVD, small vessel disease; CE, cardio embolism; ODE, other determined etiology; UDE, undetermined etiology; HBP, hypertension; DM, diabetes mellitus; AF, atrial fibrillation; sICH, symptomatic intracranial hemorrhage.

**Table S2. Baseline characteristic and outcome of patient in cohort without PS-matching**

| Characteristics | Patients without IV rtPA treatment  (n =1874) | Patients with IV rtPA treatment  (n =1462) | *P* value | SMD |
| --- | --- | --- | --- | --- |
| Age, year, mean (SD) | 60.4(13.8) | 58.4 (12.6) | <0.001^d^ | **0.149** |
| Male, n (%) | 1346 (71.8) | 1096 (75.0) | 0.043^a^ | 0.072 |
| Myocardial infarction, n (%) | 34 (1.80) | 26 (1.80) | 1.000^a^ | 0.003 |
| History of AIS, n (%) | 356 (19.0) | 204 (14.0) | <0.001^a^ | **0.137** |
| Baseline NIHSS, median (IQR) | 1 (0, 3) | 3 (1, 4) | <0.001^c^ | **0.610** |
| 0-1, n (%) | 964 (51.4) | 366 (25.0) |  |  |
| 2-3, n (%) | 597 (31.9) | 666 (45.6) |  |  |
| 4-5, n (%) | 313 (16.7) | 430 (29.4) |  |  |
| Alcohol drinking, n (%) | 306 (16.3) | 271 (18.5) | 0.072^a^ | 0.065 |
| Smoking, n (%) | 656 (35.0) | 522 (35.7) | 0.672^a^ | 0.016 |
| Baseline SBP, mmHg, mean (SD) | 151 (23.8) | 150 (22.2) | 0.216^d^ | 0.044 |
| Baseline DBP, mmHg, mean (SD) | 89.5 (15.1) | 89.4 (14.8) | 0.982^d^ | 0.001 |
| Premorbid mRS =1(n, %) | 651 (34.7) | 358 (24.5) | <0.001^a^ | **0.226** |
| TOAST |  |  | 0.027^a^ | **0.119** |
| LAA, n (%) | 767 (40.1) | 571 (39.1) |  |  |
| SVD, n (%) | 738 (39.4) | 599 (41.0) |  |  |
| CE, n (%) | 90 (4.80) | 67 (4.58) |  |  |
| ODE, n (%) | 67 (3.58) | 72 (4.92) |  |  |
| UDE, n (%) | 76 (4.01) | 89 (6.09) |  |  |
| HBP, n (%) | 1385 (73.9) | 1017 (69.6) | 0.006^a^ | 0.097 |
| DM, n (%) | 504 (26.9) | 381 (26.1) | 0.616^a^ | 0.019 |
| AF, n (%) | 47 (2.50) | 39 (2.70) | 0.858^a^ | 0.010 |
| sICH, n (%) | 10 (0.50) | 18 (1.20) | 0.045^a^ | 0.075 |
| Hospital stays, median (IQR), days | 7.93 (5.74, 11.0) | 8.00 (5.83, 11.4) | 0.301^c^ | 0.049 |
| Mortality, n (%) | 1 (0.05) | 3 (0.21) | 0.325^b^ | 0.042 |
| Excellent outcome, n (%) | 1642 (87.6) | 1245 (85.2) | 0.044^a^ | 0.072 |

^a^ Pearson’s χ^2^ test, ^b^ Fisher’s exact test, ^c^ Mann-Whitney U test, ^d^ t-test.

Abbreviation: PS, Propensity scores; IV, Intravenous; rtPA, recombinant tissue plasminogen activator antigen; SMD, standardized differences of the mean; AIS, acute ischemic stroke; NIHSS, National Institute of Health Stroke Scale; SBP, systolic blood pressure; DBP, diastolic blood pressure; ; mRS, modified Rankin Scale; TOAST, Trial of ORG 10172 in Acute Stroke Treatment; LAA, large artery atherosclerosis; SVD, small vessel disease; CE, cardio embolism; ODE, other determined etiology; UDE, undetermined etiology; HBP, hypertension; DM, diabetes mellitus; AF, atrial fibrillation; sICH, symptomatic intracranial hemorrhage.

**Table S3.** Distribution of variables with missing data before and after MI

| [Variable](javascript:;)s | Number with missing data n (%) | Cohort of PS- matching | Cohort of PS- matching +MI | *P* value |
| --- | --- | --- | --- | --- |
| Baseline SBP, mmHg, mean (SD) | 42 (1.80) | 150 (22.9) | 150 (23.0) | 0.965 |
| Baseline DBP, mmHg, mean (SD) | 45 (1.90) | 89.4 (15.0) | 89.3 (15.0) | 0.811 |
| Smoking, n (%) | 72 (3.10) | 814 (36.1) | 832 (35.8) | 0.832 |
| Alcohol drinking, n (%) | 69 (3.00) | 408 (18.1) | 418 (18.0) | 0.956 |
| TOAST | 119 (5.10) |  |  | 1.000 |
| LAA, n (%) |  | 978 (44.3) | 1026 (44.1) |  |
| SVD, n (%) |  | 887 (40.2) | 941 (40.5) |  |
| CE, n (%) |  | 117 (5.30) | 125 (5.40) |  |
| ODE, n (%) |  | 105 (4.80) | 109 (4.70) |  |
| UDE, n (%) |  | 120 (5.40) | 125 (5.40) |  |

Abbreviation: MI, multiple imputation, PS, propensity scores; SBP, systolic blood pressure; DBP, diastolic blood pressure; LAA, atherosclerosis; SVD, small vessel disease; CE, cardio embolism; ODE, other determined etiology; UDE, undetermined etiology.

Table S4. Baseline characteristic of patients before and after PS matched in subgroup of baseline NIHSS 0- 1.

|  | Cohort before PS- Matched | | | | Cohort after PS- Matched | | | |
| --- | --- | --- | --- | --- | --- | --- | --- | --- |
| [Variable](javascript:;)s | Non-IV-tPA (n= 964) | IV-tPA (n=366) | *P* | SMD | Non-IV-tPA (n= 344) | IV-tPA (n= 344) | *P* | SMD |
| Age, year, mean (SD) | 60.0 (13.85) | 57.8 (12.2) | 0.005^d^ | **0.177** | 58.0 (13.6) | 58.0 (12.2) | 0.911^d^ | 0.009 |
| Male, n (%) | 692 (71.8) | 262 (71.6) | 0.997^a^ | 0.004 | 253 (73.5) | 246 (71.5) | 0.608^a^ | 0.046 |
| Myocardial infarction, n (%) | 21 (2.20) | 11 (3.00) | 0.503^a^ | 0.052 | 10 (2.90) | 8 (2.30) | 0.811^a^ | 0.036 |
| History of AIS, n (%) | 167 (17.4) | 48 (13.1) | 0.072^a^ | **0.119** | 45 (13.1) | 45 (13.1) | 1.000^a^ | < 0.001 |
| Baseline NIHSS, median (IQR) | 0 (0, 1) | 1 (1, 1) | < 0.001^c^ | **0.692** | 1 (0, 1) | 1 (0, 1) | 0.861^c^ | 0.013 |
| Alcohol drinking, n (%) | 161 (16.9) | 64 (18.1) | 0.675^a^ | 0.031 | 59 (17.4) | 62 (18.7) | 0.743^a^ | 0.033 |
| Smoking, n (%) | 339 (35.9) | 121 (34.4) | 0.645^a^ | 0.033 | 129 (38.4) | 115 (34.7) | 0.369^a^ | 0.076 |
| Baseline SBP, mmHg, mean (SD) | 151 (23.8) | 148 (21.9) | 0.030^d^ | **0.137** | 149 (24.5) | 148 (21.6) | 0.346^d^ | 0.073 |
| Baseline DBP, mmHg, mean (SD) | 89.2 (15.0) | 88.9 (14.8) | 0.760^d^ | 0.019 | 89.6 (15.3) | 88.8 (14.8) | 0.465^d^ | 0.057 |
| Premorbid mRS =1（%） | 320（34.8%） | 114 (32.9%) | 0.585^a^ | 0.039 | 112 (32.6) | 114 (33.1) | 0.935^a^ | 0.012 |
| TOAST |  |  | 0.173^a^ | **0.156** |  |  | 0.375^a^ | **0.163** |
| LAA, n (%) | 308 (35.5) | 111 (32.0) |  |  | 106 (33.5) | 109 (33.4) |  |  |
| SVD, n (%) | 428 (49.3) | 174 (50.1) |  |  | 147 (36.5) | 157 (48.2) |  |  |
| CE, n (%) | 46 (5.30) | 14 (4.00) |  |  | 24 (7.60) | 13 (4.00) |  |  |
| ODE, n (%) | 42 (4.80) | 19 (5.50) |  |  | 16 (5.10) | 19 (5.80) |  |  |
| UDE, n (%) | 44 (5.10) | 29 (8.40) |  |  | 23 (7.30) | 28 (8.60) |  |  |
| HBP, n (%) | 692 (71.8) | 246 (67.2) | 0.117^a^ | 0.099 | 229 (66.6) | 232 (67.4) | 0.871^a^ | 0.019 |
| DM, n (%) | 254 (26.3) | 79 (21.6) | 0.085^a^ | **0.112** | 72 (20.9) | 74 (21.5) | 0.926^a^ | 0.014 |
| AF, n (%) | 23 (2.40) | 6 (1.60) | 0.534^a^ | 0.053 | 4 (1.20) | 6 (1.7) | 0.752^b^ | 0.071 |
| sICH, n (%) | 1 (0.10) | 1 (0.30) | 0.475^b^ | 0.039 | 1 (0.30) | 1 (0.30) | 1.000^b^ | < 0.001 |
| Hospital stays, median (IQR), days | 7.56 (5.58, 10.07) | 7.69 (5.07, 9.99) | 0.948^c^ | 0.036 | 7.73 (5.72, 9.98) | 7.83 (5.46, 10.06) | 0.757^c^ | 0.071 |
| Mortality, n (%) | 0 (0.0) | 0 (0.0) | NA | < 0.001 | 0 (0.0) | 0 (0.0) | NA | < 0.001 |
| Excellent outcome, n (%) | 913(94.7) | 336(91.8) | 0.064^a^ | **0.116** | 319 (92.7) | 315 (91.6) | 0.671^a^ | 0.043 |

^a^ Pearson’s χ^2^ test, ^b^ Fisher’s exact test, ^c^ Mann-Whitney U test, ^d^ t-test.

Abbreviation: PS, Propensity scores; IV, Intravenous; rtPA, recombinant tissue plasminogen activator antigen; SMD, standardized differences of the mean; AIS, acute ischemic stroke; NIHSS, National Institute of Health Stroke Scale; SBP, systolic blood pressure; DBP, diastolic blood pressure; mRS, modified Rankin Scale; TOAST, Trial of ORG 10172 in Acute Stroke Treatment; LAA, large artery atherosclerosis; SVD, small vessel disease; CE, cardio embolism; ODE, other determined etiology; UDE, undetermined etiology; HBP, hypertension; DM, diabetes mellitus; AF, atrial fibrillation; sICH, symptomatic intracranial hemorrhage; NA, not application.

Table S5. Baseline characteristic of patients before and after PS matched in subgroup of baseline NIHSS 2- 3.

|  | Cohort before PS- Matched | | | | Cohort after PS- Matched | | | |
| --- | --- | --- | --- | --- | --- | --- | --- | --- |
| [Variable](javascript:;)s | Non-IV-tPA (n= 597) | IV-tPA (n=666) | *P* | SMD | Non-IV-tPA (n= 486) | IV-tPA (n= 486) | *P* | SMD |
| Age, year, mean (SD) | 60.40 (14.18) | 59.15 (12.59) | 0.097^d^ | 0.093 | 59.5 (13.9) | 58.0 (12.3) | 0.085^d^ | **0.111** |
| Male, n (%) | 427 (71.5) | 497 (74.6) | 0.239^a^ | 0.070 | 353 (72.6) | 354 (72.8) | 1.000^a^ | 0.005 |
| Myocardial infarction, n (%) | 8 (1.30) | 10 (1.50) | 0.997^a^ | 0.014 | 5 (1.00) | 6 (1.20) | 1.000^a^ | 0.019 |
| History of AIS, n (%) | 123 (20.6) | 88 (13.2) | 0.001^a^ | **0.199** | 79 (16.3) | 83 (17.1) | 0.796^a^ | 0.022 |
| Baseline NIHSS, median (IQR) | 2 (2, 3) | 2 (2, 3) | 0.018^c^ | **0.134** | 2 (2, 3) | 2 (2, 3) | 0.745^c^ | 0.021 |
| Alcohol drinking, n (%) | 96 (16.5) | 129 (20.1) | 0.122^a^ | 0.093 | 83 (17.5) | 95 (20.2) | 0.336^a^ | 0.068 |
| Smoking, n (%) | 211 (36.9) | 235 (36.7) | 0.982^a^ | 0.005 | 170 (36.3) | 171 (36.4) | 1.000^a^ | 0.001 |
| Baseline SBP, mmHg, mean (SD) | 152 (23.3) | 150 (21.7) | 0.375^d^ | 0.050 | 152 (23.7) | 152 (21.8) | 0.815^d^ | 0.015 |
| Baseline DBP, mmHg, mean (SD) | 90.2 (14.8) | 88.9 (14.7) | 0.122^d^ | 0.088 | 90.6 (15.0) | 90.0 (14.9) | 0.561^d^ | 0.038 |
| Premorbid mRS =1（%） | 225 (39.5%） | 177 (28.4%) | <0.001^a^ | 0.235 | 171 (35.2) | 172 (35.4) | 1.000^a^ | 0.004 |
| TOAST |  |  | 0.012^a^ | **0.209** |  |  | 0.145^a^ | **0.172** |
| LAA, n (%) | 290 (50.6) | 276 (43.3) |  |  | 229 (49.1) | 211 (45.5) |  |  |
| SVD, n (%) | 222 (38.7) | 266 (41.7) |  |  | 189 (40.6) | 185 (39.9) |  |  |
| CE, n (%) | 25 (4.40) | 24 (3.80) |  |  | 18 (3.90) | 16 (3.40) |  |  |
| ODE, n (%) | 17 (3.00) | 32 (5.00) |  |  | 12 (2.60) | 22 (4.70) |  |  |
| UDE, n (%) | 19 (3.30) | 40 (6.30) |  |  | 18 (3.90) | 30 (6.50) |  |  |
| HBP, n (%) | 458 (76.7) | 458 (68.8) | 0.002^a^ | **0.179** | 365 (75.1) | 357 (73.5) | 0.607^a^ | 0.038 |
| DM, n (%) | 437 (73.2) | 487 (73.1) | 1.000^a^ | 0.002 | 360 (74.1) | 381 (78.4) | 0.132^a^ | **0.102** |
| AF, n (%) | 13 (2.20) | 22 (3.30) | 0.296^a^ | 0.069 | 10 (2.10) | 14 (2.90) | 0.535^a^ | 0.053 |
| sICH, n (%) | 6 (1.00) | 8 (1.20) | 0.095^a^ | 0.019 | 4 (0.80) | 4 (0.80) | 1.000^b^ | < 0.001 |
| Hospital stays, median (IQR), days | 8.28 (5.81, 11.66) | 8.00 (5.84, 11.49) | 0.728^c^ | 0.067 | 8.26 (5.69, 11.53) | 8.04 (5.93, 11.02) | 0.815^c^ | 0.066 |
| Mortality, n (%) | 1 (0.2) | 2 (0.3) | 1.000^b^ | 0.027 | 0 (0.0) | 0 (0.0) | NA | < 0.001 |
| Excellent outcome, n (%) | 506 (84.8) | 573 (86.0) | 0.573^a^ | 0.036 | 410 (84.4) | 425 (87.4) | 0.197^a^ | 0.089 |

^a^ Pearson’s χ^2^ test, ^b^ Fisher’s exact test, ^c^ Mann-Whitney U test, ^d^ t-test.

Abbreviation: PS, Propensity scores; IV, Intravenous; rtPA, recombinant tissue plasminogen activator antigen; SMD, standardized differences of the mean; AIS, acute ischemic stroke; NIHSS, National Institute of Health Stroke Scale; SBP, systolic blood pressure; DBP, diastolic blood pressure; mRS, modified Rankin Scale; TOAST, Trial of ORG 10172 in Acute Stroke Treatment; LAA, large artery atherosclerosis; SVD, small vessel disease; CE, cardio embolism; ODE, other determined etiology; UDE, undetermined etiology; HBP, hypertension; DM, diabetes mellitus; AF, atrial fibrillation; sICH, symptomatic intracranial hemorrhage; NA, not application.

Table S6. Baseline characteristic of patients before and after PS matched in subgroup of baseline NIHSS 4- 5.

|  | Cohort before PS- Matched | | | | Cohort after PS- Matched | | | |
| --- | --- | --- | --- | --- | --- | --- | --- | --- |
| [Variable](javascript:;)s | Non-IV-tPA (n= 313) | IV-tPA (n=430) | *P* | SMD | Non-IV-tPA (n= 241) | IV-tPA (n= 241) | *P* | SMD |
| Age, year, mean (SD) | 61.2 (14.5) | 57.7 (12.8) | 0.001^d^ | **0.256** | 60.5 (13.7) | 61.1 (12.5) | 0.591^d^ | 0.049 |
| Male, n (%) | 227 (72.5) | 337 (78.6) | 0.070^a^ | **0.141** | 182 (75.5) | 172 (71.4) | 0.353^a^ | 0.094 |
| Myocardial infarction, n (%) | 5 (1.60) | 5 (1.20) | 0.849^a^ | 0.038 | 3 (1.20) | 3 (1.20) | 1.000^a^ | <0.001 |
| History of AIS, n (%) | 66 (21.2) | 68 (15.9) | 0.079^a^ | **0.137** | 48 (19.9) | 46 (19.1) | 0.908^a^ | 0.021 |
| Baseline NIHSS, median (IQR) | 4 (4, 5) | 4 (4, 5) | 0.001^c^ | **0.242** | 4 (4, 5) | 4 (4, 5) | 0.160^c^ | **0.128** |
| Alcohol drinking, n (%) | 49 (16.4) | 78 (18.8) | 0.477^a^ | 0.062 | 41 (17.7) | 44 (18.9) | 0.845^a^ | 0.029 |
| Smoking, n (%) | 106 (35.6) | 166 (39.6) | 0.306^a^ | 0.084 | 84 (36.2) | 86 (36.4) | 1.000^a^ | 0.005 |
| Baseline SBP, mmHg, mean (SD) | 150 (24.5) | 151 (23.3) | 0.596^d^ | 0.04 | 150 (24.6) | 151 (23.8) | 0.630^d^ | 0.044 |
| Baseline DBP, mmHg, mean (SD) | 88.9 (16.2) | 90.8 (14.8) | 0.115^d^ | **0.118** | 89.0 (16.3) | 90.4 (15.3) | 0.344^d^ | 0.087 |
| Premorbid mRS =1（%） | 106 (35.7%) | 67 (16.5%) | <0.001^a^ | 0.448 | 63 (26.1) | 62 (25.7) | 1.000^a^ | 0.009 |
| TOAST |  |  | 0.019^a^ | **0.265** |  |  | 0.568^a^ | **0.16** |
| LAA, n (%) | 169 (56.9) | 184 (44.6) |  |  | 120 (52.2) | 109 (46.6) |  |  |
| SVD, n (%) | 88 (29.6) | 159 (38.5) |  |  | 78 (33.9) | 85 (36.3) |  |  |
| CE, n (%) | 19 (6.40) | 29 (7.00) |  |  | 14 (6.10) | 20 (8.50) |  |  |
| ODE, n (%) | 8 (2.70) | 21 (5.10) |  |  | 7 (3.00) | 11 (4.70) |  |  |
| UDE, n (%) | 13 (4.40) | 20 (4.80) |  |  | 11 (4.80) | 9 (3.80) |  |  |
| HBP, n (%) | 235 (75.1) | 313 (72.8) | 0.538^a^ | 0.052 | 181 (75.1) | 184 (76.3) | 0.832^a^ | 0.029 |
| DM, n (%) | 223 (71.2) | 307 (71.4) | 1.000^a^ | 0.003 | 76 (31.5) | 74 (30.7) | 0.922^a^ | 0.018 |
| AF, n (%) | 11 (3.50) | 11 (2.60) | 0.589^a^ | 0.056 | 11 (4.60) | 9 (3.70) | 0.819^a^ | 0.042 |
| sICH, n (%) | 3 (1.00) | 9 (2.10) | 0.256^b^ | 0.093 | 3 (1.20) | 5 (2.10) | 0.724^b^ | 0.065 |
| Hospital stays, median (IQR), days | 9.01 (6.49, 13.09) | 8.78 (6.39, 12.40) | 0.222^c^ | 0.061 | 9.03 (6.58, 13.1) | 8.95 (6.43, 12.7) | 0.590^c^ | 0.076 |
| Mortality, n (%) | 0 (0.00) | 1 (0.20) | 1.000^b^ | 0.068 | 0 (0.00) | 0 (0.00) | NA | < 0.001 |
| Excellent outcome, n (%) | 223 (71.2) | 336 (78.1) | 0.039^a^ | **0.159** | 163 (67.6) | 182 (75.5) | 0.069^a^ | **0.175** |

^a^ Pearson’s χ^2^ test, ^b^ Fisher’s exact test, ^c^ Mann-Whitney U test, ^d^ t-test.

Abbreviation: PS, Propensity scores; IV, Intravenous; rtPA, recombinant tissue plasminogen activator antigen; SMD, standardized differences of the mean; AIS, acute ischemic stroke; NIHSS, National Institute of Health Stroke Scale; SBP, systolic blood pressure; DBP, diastolic blood pressure; mRS, modified Rankin Scale; TOAST, Trial of ORG 10172 in Acute Stroke Treatment; LAA, large artery atherosclerosis; SVD, small vessel disease; CE, cardio embolism; ODE, other determined etiology; UDE, undetermined etiology; HBP, hypertension; DM, diabetes mellitus; AF, atrial fibrillation; sICH, symptomatic intracranial hemorrhage; NA, not application.
